# Validation of the 2 × 24 h recall method and a 7-d web-based food diary against doubly labelled water in Danish adults

**DOI:** 10.1017/S0007114523000454

**Published:** 2023-10-28

**Authors:** Anja Biltoft-Jensen, Karin Hess Ygil, Lenette Knudsen, Jeppe Matthiessen, Sisse Fagt, Ellen Trolle, Trine Holmgaard Nielsen, Diane McIntosh Hansen, Cecilie Löe Licht, Maurice Martens, Catherine Hambly, John R. Speakman, Tue Christensen

**Affiliations:** 1 National Food Institute, Technical University of Denmark, Kongens, Lyngby 2800, Denmark; 2 Steno Diabetes Center Copenhagen, Region Hovedstaden, Gentofte, Denmark; 3 Danish Cancer Society, Counselling Centre Herlev, Herlev, Denmark; 4 Kalvebod Fælled School, København S, Denmark; 5 Center for Cancer Immune Therapy (CCIT), Department of Oncology, Herlev Hospital, Herlev, Denmark; 6 Centerdata, Tilburg University, Tilburg, Netherlands; 7 Institute of Biological and Environmental Sciences, University of Aberdeen, Aberdeen, Scotland, UK; 8 Shenzhen Key Laboratory of Metabolic Health, Center for Energy Metabolism and Reproduction, Shenzhen Institutes of Advanced Technology, Chinese Academy of Sciences, Shenzhen, People’s Republic of China

**Keywords:** Dietary assessment, Misreporting, Reported energy intake, Physical activity measurement, Pedometer, Energy expenditure

## Abstract

The European Food Safety Authority has suggested that EU countries implement the 2 × 24 h diet recall (2 × 24 h DR) method and physical activity (PA) measurements for national dietary surveys. Since 2000, Denmark has used 7 d food diaries (7 d FD) with PA questionnaires and measurements. The accuracy of the reported energy intakes (EI) from the two diet methods, pedometer-determined step counts and self-reported time spent in moderate-to-vigorous PA (MVPA) were compared with total energy expenditure measured by the doubly labelled water (TEEDLW) technique and with PA energy expenditure (PAEE), respectively. The study involved fifty-two male and sixty-eight female volunteers aged 18–60 years who were randomly assigned to start with either the 24 h DR or the web-based 7 d FD, and wore a pedometer for the first 7 d and filled in a step diary. The mean TEEDLW (11·5 MJ/d) was greater than the mean reported EI for the 7 d FD (9·5 MJ/d (*P* < 0·01)) but the same as the 2 × 24 h DR (11·5 MJ/d). The proportion of under-reporters was 34 % (7 d FD) and 4 % (2 × 24 h DR). Most participants preferred the 7 d DR as it was more flexible, despite altering their eating habits. Pearson’s correlation between steps corrected for cycling and PAEE was *r* = 0·44, *P* < 0·01. Spearman’s correlation for self-reported hours spent in MVPA and PAEE was *r* = 0·58, *P* < 0·01. The 2 × 24 h DR performs better than the existing 7 d FD method. Pedometer-determined steps and self-reported MVPA are good predictors of PAEE in adult Danes.

In many European countries, national dietary surveys are conducted to monitor populations’ dietary intake and physical activity levels (PAL). Accurate and reliable dietary data are crucial for food safety, public health initiatives and sustainability. The results from the Danish National Survey of Diet and Physical activity (DANSDA) are used to investigate associations and generate new hypotheses between dietary exposures (nutrients, foods/food groups, dietary patterns, eating behaviour) or physical activity (PA) and risk factors for chronic diseases and nutrient status. Furthermore, results are used to evaluate nutrition and public health policies (compliance with food-based dietary guidelines, nutrient recommendations and PA guidelines), sustainability of diets and exposure assessment of natural toxicants, contaminants, additives and other food chemicals for risk assessment.

To obtain consistent and comparable food and nutrient data across countries and to facilitate uniform legislation on food safety, the European Food Safety Authority (EFSA) has suggested that the 2 × 24 h diet recall (2 × 24 h DR) assessment method should be implemented in EU countries^(1)^. The method includes two detailed, personal interviews (one in-person and one by telephone) to recall what and how much participants ate and drank within the last 24 h. Dietary data are collected with a computerised methodology with a high level of quality assurance for consistency, and capabilities to capture detailed food descriptors. The method also includes a short, non-quantitative food propensity questionnaire which records the frequency of intake of episodically consumed foods covering all seasons during the past year. In addition, information on participants’ weight, height and PAL should also be collected during the survey. The objective of PA assessment is to rank individuals into low, medium and high PAL categories, without exact PAL values. This information should be used to correct for PAL when calculating cut-off points for energy intake (EI) misreporting as suggested by Black^(1,2)^. The method described by EFSA in their guidance on the EU Menu methodology, that is, 2 × 24 h DR, has been validated using fatty acids in phospholipids and serum carotenoids as fish and fruit and vegetable intake biomarkers, respectively^(1,3)^. The method has not compared reported EI to total energy expenditure (TEE_DLW_) measured with the doubly labelled water (DLW) technique which should match if the person is in energy balance.

In Denmark, a 7-d self-administered food diary (7 d FD) has been applied since 1995, and PA measurements (questionnaire and/or pedometry and a 7-d step diary) have been included since 2000 in the DANSDA^(4)^. Steps measured with a pedometer are an inexpensive, easily measurable, interpretable and communicable metric of the total amount of daily ambulatory activity^(5)^. However, validation of pedometers as indicators of PA in a sample of adult Danes is lacking.

The DANSDA method includes a food diary, which participants fill in for seven consecutive days. The 7 d FD has been developed into a web-based food diary that contains a comprehensive food list, where all portion sizes are estimated by a series of photos of commonly eaten food items. The web-based food diary has been validated in 8–11-year-old children for EI against TEE (estimated by accelerometers)^(6)^, blood carotenoids as markers of fruit and vegetable intake^(7)^, *n*-3 fatty acids EPA + DHA as markers of fish intake^(8)^ and alkylresorcinols as markers of whole-grain intake^(9)^. However, the web-based food diary has not been validated in adults.

The suggested 2 × 24 h DR is a retrospective assessment method, which may minimise the risk of changes in dietary behaviour caused by registration itself. However, the 2 × 24 h DR is known to be prone to socially desirable answers (if interviewer-administered) and depend on the participant’s memory^(10)^. As the potential errors between methods are different and depend on specific methodological details, implementing a new national dietary assessment method requires validation and comparison with the previously applied method concerning the validity of food reporting. Information on the magnitude of reporting error is necessary for the interpretation of national dietary survey data for food safety and health promotion, and possible comparisons with previous dietary surveys. Factors such as acceptability in the population are also important given a decreasing response rate in national dietary surveys across Europe^(11)^.

The primary objective of the present study was to use TEE measured by the DLW technique as a biomarker to validate EI estimated by both the self-administered web-based 7 d FD and the interviewer administered 2 × 24 h DR methods and to assess participant acceptability of the two diet registration methods. Furthermore, secondary objectives were to examine if adding a third recall provides an additional improvement in EI estimates and evaluate pedometer-determined steps and self-reported moderate-to-vigorous PA (MVPA) during leisure as predictors of PA energy expenditure (PAEE).

## Methods

### Participants

The study was conducted at the National Food Institute (NFI), Technical University of Denmark. One hundred and twenty volunteers aged 18–60 years were recruited through advertisement in the university newspaper, on the university’s local network (students and employees), and newsletter, in local newspapers and through Facebook. Interested participants conducted an on-line preliminary screening about age, Danish language, Internet access, weight stability and chronic diseases and signed up for the study through a website. Participants were chosen to get an equal distribution of men and women in the age groups 18–30, 31–45 and 46–60 years to capture the different life stages in the adult population. All participants underwent an in-depth telephone screening interview. Inclusion criteria were Danish speakers, access to the Internet, acceptance of the participant tasks involved, basic good health (no chronic disease requiring medicine), weight stable and not actively trying to lose weight or taking medications known to affect food and EI, appetite or water balance. Pregnant and lactating females and nutrition professionals were also excluded.

Participants were informed about the study through information meetings, during the screening interviews and written information was sent twice to all participants. Everyone entering the study provided written informed consent. This study was conducted according to the guidelines laid down in the Declaration of Helsinki, and all procedures involving human subjects were approved by the Regional Ethical Committee of Copenhagen and by the Danish Data Protection Agency (no 17006825).

### Study design

The study was designed as a cross-over study. Within sex and age groups, half (*n* 60) of the population was randomly assigned to start with either the 24 h DR or the 7 d FD method. Data were collected during September and October 2017.

**Fig. 1.**
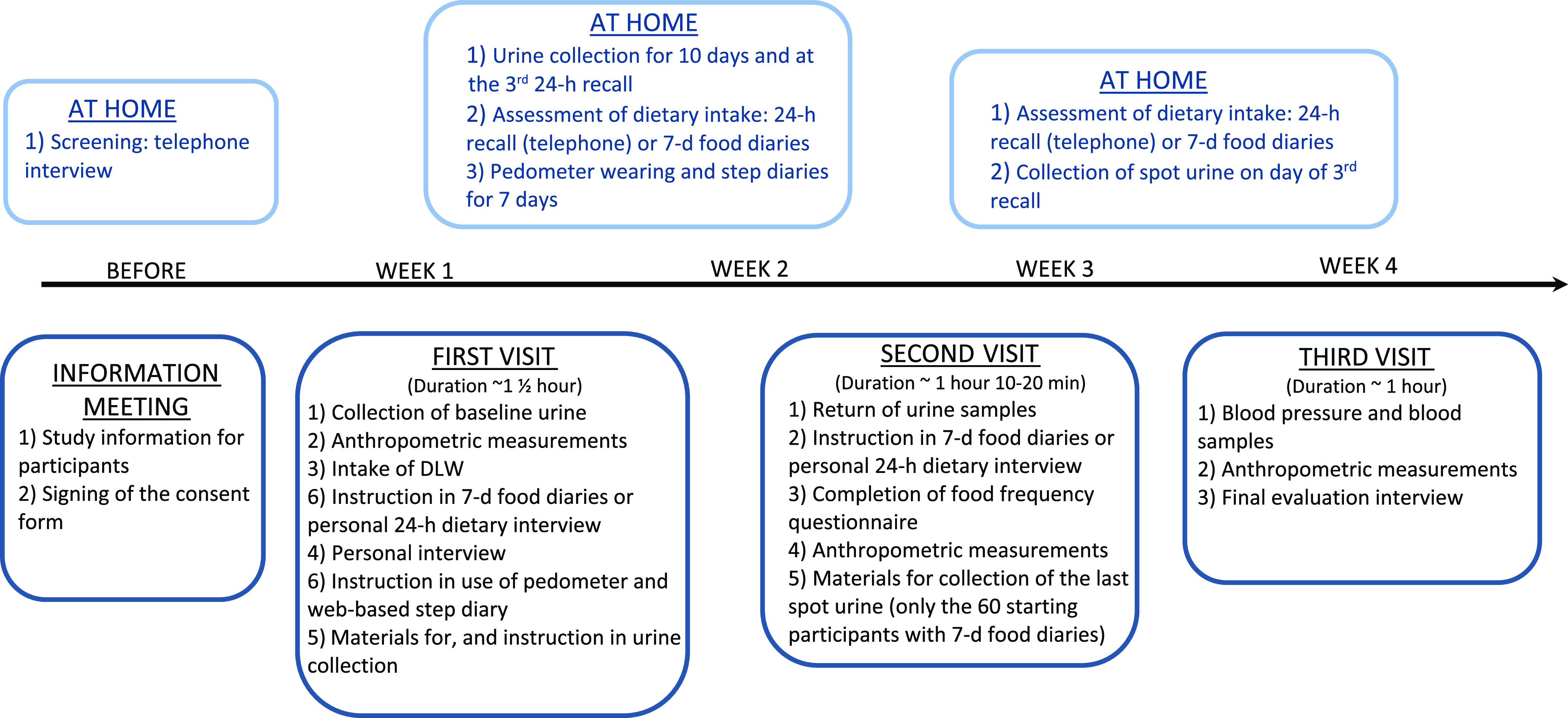
Flow chart of the study design, measurement of dietary intake, pedometry, energy expenditure, anthropometry, blood samples and blood pressure.

A flow chart of the study design is illustrated in Fig. 1. Each subject entered the study for an approximately 4-week period of free-living activities, and data were collected during three centre visits (CV) at the NFI and at home, including two scheduled telephone 24 h DR, diet recording and PA registration for 7 d and spot urine sampling for 11 d.

Anthropometric measurements of weight, height, waist circumference and body composition were taken at all three CV. At CV1, all participants had their height measured, provided a background urine sample, drank a dose of DLW and were instructed in urine sample collection at home. Furthermore, social background characteristics such as education, household composition, smoking and leisure time spent on MVPA during the last week were ascertained by a short interview, and participants were instructed in how to wear the pedometers and fill in the web-based step diary.

A personal 24 h DR was conducted at CV1 or CV2 by trained interviewers and detailed instruction on recording of dietary intake in the 7 d FD was provided individually to all participants at CV1 or CV2 depending on the starting method. The blood sampling, blood pressure and an evaluation questionnaire were completed at CV3. The 3 × 24 h DR were completed at randomly selected weekdays, covering all 7 week days at a group level and at least 1 week apart as recommended by EFSA^(1)^.

### 24 h diet recall: the automated multiple pass method

The Food Surveys research group of the United States Department of Agriculture (USDA) has developed an automated multiple pass method (AMPM) for conducting 24 h recall, to ensure accuracy in the collection of dietary recall data by automating the interview with computerised questions, prompts and details about reported foods. The AMPM has the advantage that it has been used for the collection of dietary intake data for many years and undergoes an update every other year^(12)^. The AMPM has been validated against DLW in 524 American adult volunteers (30–69 years)^(13)^. The results showed an underestimation of reported EI by 11 % in the total population, and <3 % in participants with normal weight.

The AMPM contains a five-step approach beginning with a quick list where respondents report all foods consumed in the prior 24 h period. The second step includes a series of questions that probe for foods that are commonly forgotten during step one. The third step collects the time each food was eaten and the name of the eating occasion. At the fourth step, descriptions are obtained for each food reported, along with quantities consumed and where the food was obtained. All foods in the instrument belonged to one of over 100 food categories of similar foods (e.g. bread, sandwiches, pasta, milk). The AMPM prompted different detail questions about a food depending on what category it belonged to. Common details captured by the instrument included the source (e.g. homemade), preparation (e.g. cooking method, type of fat or liquid added), brand names and anything added to the food.

The fifth step is a final review question, which provides the respondent a last opportunity to recall any foods that had not been reported previously in the interview^(12)^. Six trained interviewers with a formal nutrition, public health or biology education background conducted all 24 h DR interviews (358 interviews in total).

The AMPM was versioned into Danish AMPM including Danish food lists and cooking practices. The translation process of the AMPM was managed with the Translation Management Tool developed by the Centerdata at Tilburg University in the Netherlands^(14)^. The amount consumed was estimated using metrics (g, ml, l), spoons, pieces, small, medium, large and USDA’s measuring guide Food Model Booklet (FMB)^(15)^. The FMB was also versioned into Danish Measuring Guides including images of Danish plates, bowls, cups, mugs and glasses. All participants received the FMB to take home for the 24 h DR telephone interviews.

### 7-d food diaries

Self-reported EI was assessed for all individuals using a web-based 7 d FD where participants recorded their food intake each day for 7 consecutive days. The 7 d FD guides respondents through six daily eating occasions (breakfast, morning snack, lunch, afternoon snack, dinner and evening snack). For the diet records, a database of 1340 beverages, food items and dishes was available, either through category browsing or through free text search, aided by a spellcheck application. It was also possible to type in foods not otherwise found through category browsing or a text search. The amount consumed was estimated by selecting the portion size from four different digital images among 320 photo series. The 7 d FD included internal checks for frequently forgotten foods (spreads, sugar, sauces, dressings, snacks, candy and beverages). Foods were reported as eaten on a generic level with no details of brand names, cooking method, types of fat used in cooking, etc. Standard recipes were used to estimate EI.

If a participant failed to report for a day, they were reminded the next day by an e-mail. If this did not help, participants received a reminder phone call the day after.

For participants to be included in the analyses, the7 d FD had to be completed for at least 4 d, including 1 weekend day to represent a weekly recording.

For both methods, the EI was calculated for each individual with the use of the software system GIES (Version 1.000 i5 – 2014-09-10), developed at the NFI, Technical University of Denmark and the Danish Food Composition Databank Frida (version 3; Søborg, Denmark; 23-03-2018).

### Measurement of total energy expenditure using doubly labelled water

TEE was determined by using the DLW method^(16,17)^. At CV1 at the start of data collection, between the hours of 08·00–17·00, participants provided a baseline urine sample into a 30 ml tube to assess naturally occurring levels of isotopes. They were then weighed to the nearest 0·1 kg (Precision Health Scale UC-351PBT-Ci, A&D Medical). Their weight was used to calculate the appropriate dose (weighed to 4 decimal places) of pre-mixed DLW according to Speakman^(18)^. The dose contained 5 % ^2^H and 10 % O^18^. To ensure the whole dose was consumed, the glass vials were refilled with additional water which participants were asked to consume and the time of dosing recorded. Participants were instructed to collect approximately 30 ml urine samples from the second void each morning for 10 d and a final sample on day 21 (to cover the 3 × 24 h period) and to record the date and time of collection. Participants were provided with cooler bags with freezer elements and racks to store the urine samples in their home freezers until they could deliver them to the centre. Participants with no available freezers delivered their samples to the centre on daily basis.

All urine samples were stored at −18°C at the centre, before being aliquoted into 2·0 ml cryotube vials and frozen at −80°C. Once collection was complete, they were sent for analysis to the Energetics Research Group, University of Aberdeen.

Analysis of the isotopic enrichment of urine was performed blind, using a Liquid Isotope Water Analyser (Los Gatos Research)^(19)^. The urine was vacuum distilled^(20)^, and the resulting distillate was used for analysis. Samples were run alongside five lab standards for each isotope and three International standards to adjust for daily variation and correct delta values to ppm. Daily isotope enrichments were log_e_ converted and the elimination constants (k_o_ and k_d_) were calculated by fitting a least squares regression model to the log_e_ converted data. The back extrapolated intercept was used to calculate the isotope dilution spaces (N_o_ and N_d_). A two-pool model, specifically Speakman, was used to calculate rates of CO_2_ production^(21)^.

### Measurement of physical activity energy expenditure

PAEE was determined by subtracting BMR, calculated according to Henry^(22)^ based on height and weight, from TEE_DLW_.

### Anthropometric measurements

The height of the participant was measured with 0·1 cm accuracy using a wall-mounted stadiometer (Kern MSF 200). Two measurements were performed for each participant. If the two measurements deviated more than 1 cm, a third measurement was taken, and the mean of three measurements were used.

Weight and body composition was measured using a bioimpedance analysis (Tanita BC 418 MA), wearing only light clothing and without shoes. Trained staff carried out all anthropometric measurements.

### Definition of mis-reporters and accurate reporters

Since day-to-day variation in EI and in EE is normal, exact agreement between EI and TEE over a short dietary assessment period in one individual is unlikely. Therefore, the accuracy of the reported EI was assessed using the confidence limits of agreement for EI:TEE_DLW_ as suggested by Black and Cole^(23)^. Participants were classified as acceptable reporters (AER), under-reporters (UER) or over-reporters (OER) according to whether the individual’s EI:EE ratio was within, below or above the 95 % confidence limits of agreement between the two measurements. The 95 % confidence limits of agreement between EI_2 × 24 h DR,_ EI_3 × 24 h DR_ or EI_7-d FR_ and TEE_DLW_ were calculated as 95 % CL = ± 2 × √(CV^2^_TEE_ + (CV^2^_EI_/d)), where d is the number of days of assessment and CV_EI_ and CV_TEE_ are the pooled mean within subject coefficients of variation in EI by 2 × 24 h DR (24 %), 3 × 24 h DR (28 %) or 7 d FR (28 %) and TEE_DLW_ (8·2 %), respectively. For the 7 d FD, the number of days was 7, and for 2- and 3 × 24 DR, the number of days was 2 and 3, respectively. For CV_TEE_ estimated by the DLW technique, we used the intra-individual CV proposed by Black and Cole as only single measurements of TEE_DLW_ were taken^(23)^. Black and Cole evaluated data from twenty-one reports of repeated trials of DLW data collection. For the 7 d FD, participants were considered UER with EI:TEE_DLW_ < 0·73, and as OER at EI:TEE_DLW_ > 1·27. For 2 × 24 h DR, participants were considered UER with EI:TEE_DLW_ < 0·62, and OER at EI:TEE_DLW_ > 1·38. For the 3 × 24 h DR, participants were considered UER with EI:TEE_DLW_ < 0·64, and as OER at EI:TEE_DLW_ > 1·36.

### Physical activity

PA was measured by a pedometer (Yamax SW 200) worn for 7 consecutive days. The pedometer-determined measurements in this study were steps per day. Participants were instructed in person how to wear and use the pedometer. A web-based step diary, that is, a software programme useable from a computer, tablet or mobile, was used to record the number of steps taken each day together with wear time, cycling time and time spent in sports activities. At least four valid days defined as ≥10 h/d of wear, and steps between 100 and 50 000 steps/d of monitoring were required for an individual to be included in the analysis to assess the habitual level of PA. Average steps/d <1000 or >25 000 for a monitoring frame of 4–7 d were treated as outliers and were excluded unless the low or high daily step counts could be verified by low or high levels of daily ambulatory activity in the step diaries^(24)^. As cycling was the most frequently reported non-ambulatory activity, whereas sports and exercise activities such as swimming, weight training and horseback riding were less common, step equivalents from cycling were taken into consideration as described by Matthiessen *et al.*^(24)^.

Furthermore, the background interview included questions on PA during leisure and on leisure time spent on MVPA during the last week from the validated Nordic Physical Activity Questionnaire (NPAQ)^(25,26)^. NPAQ may be used to assess compliance with the PA guidelines as it covers both time and intensity.

### Evaluation questionnaire, focus group and interviewer evaluation

All participants completed a short dietary assessment evaluation questionnaire containing fourteen questions with closed and open answer categories at CV3. A focus group was conducted by a member of the research team with training and experience in qualitative research 2 weeks after the study was completed. The focus group was conducted to get more insight into participants’ experiences with, and reactions to, the data collection tools used in the study. The focus group consisted of three males and three females between 18 and 60 years with different educational levels, and who completed both methods. The aim of the questionnaire and the focus group was to learn more about factors that influenced the reported food intake. Both the questionnaire and the focus group focused on the same aspects of the dietary assessment with the two methods:

- Food reporting: remembering items, willingness to report, change of eating habits and satisfaction with detail level
- Adequacy and ease of portion size estimation
- Participant burden
- Which method reflected dietary habits best

The focus groups were taped, transcribed, themed and synthesised.

All interviewers attended an evaluation meeting. The meeting was based on a set of questions that provided a base for discussing the interviewers’ challenges and experiences of the AMPM interviewer system and the interviews themselves with the participants. The interviewers’ answers and remarks were noted by three people. The answers were thereafter transcribed, themed and synthesised.

### Statistics

Sample size calculation was based on results from a similar previous American study^(13)^. The log sd of the log difference between EI assessed with 3 × 24 h DR and TEE measured by the DLW method was 0·36. With a 10 % difference between EI and TEE and a significance level of 5 % and a power of 80 %, ninety-four persons were needed for the present study. To allow for dropouts, 120 persons were aimed for.

Normality plots and the Kolmogorov–Smirnov test were performed to assess skewness of distributions. The distributions of EI and TEE were approximately normally distributed, that is, data followed roughly a normal distribution. Paired *t* tests were used to test differences between EI and TEE. To describe the direction and strength of the linear relationship between EI and TEE, and PAEE and steps including cycling (StepsCYCLING) Pearson’s correlation coefficients were calculated. The Spearman’s rank correlation coefficients were used to test the direction and strength of the relationship between MVPA and PAEE. BMR was calculated after Henry based on height and weight^(22)^. Differences between correlations were tested according to Meng *et al*.^(27)^ Bland–Altman plots were created for illustrating the difference between EI and TEE, and the mean of the two. To visualise agreement between usual EI estimated from the 2 × 24 h DR and TEE, we used the multiple source method (MSM) to estimate usual intakes including the explanatory variables ‘age’, ‘sex’ and the interaction term ‘age × sex’^(28,29)^. This model removes the effect of day-to-day variability in the 2 × 24 h DR mean estimates.

Agreement on category level between TEE and EI, and PAEE and Steps_CYCLING_ and hours spent in MVPA per week was examined by classification of EI and TEE and PAEE and Steps_CYCLING_ into tertiles. Through a cross-tabulation, the Cohen’s weighed *κ* was obtained. All statistical analysis was performed in SPSS, version 25, IBM Corp., 2017.

## Results

One hundred and twenty subjects completed the study. Two subjects only had one telephone interview and thus two 24 h DR in total. Another three subjects completed all three 24 h DR, but three of their single 24 h DR were lost due to a power failure. Therefore, 115 participants had 3 × 24 h DR and 120 participants had 2 × 24 h DR. Each participant completed all 7 d FDs. One subject was categorised as an outlier with the 7 d FD, and two and one outliers were observed with the 2 × 24 h DR and the 3 × 24 h DR, respectively (EI – TEE > 3 SD away from the mean difference). However, excluding the outliers did not change the results significantly, so we decided to keep the outliers in the analysis.

### Characteristics of the study population

General characteristics of the study population are presented in Table 1. The mean age of the study population was 39 years (sd 12) and 38% of both males (48 %) and females (31 %) had a BMI over 25 kg/m^2^. Sixty percentage of both males (73 %) and females (49 %) engaged in sports activities or hard exercise several times a week. Sixty-four percentage of both males (58 %) and females (69 %) had a medium or a long (15+ years) higher education. Only eight participants had no or a vocational education or similar. They were included in short education (short education = no education after school, vocational training, shorter courses and short-term higher education).

**Table 1.** Characterisation of study population (Mean values and standard deviations)

Study population (*n* 120)	All		Males		Females	
	Mean	sd	Mean	sd	Mean	sd
Sex, age and weight						
Sex	–		43		57	
Age (years)	38·8	12·0	37·4	13·0	40·0	11·0
Weight (kg)	73·5	12·4	81·5	10·2	67·3	10·2
Weight status						
Underweight	2		0		3	
Normal weight	60		52		66	
Overweight	33		44		25	
Obese	5		4		6	
Leisure time physical activity						
Exercises hard and competes several times a week	17		27		9	
Does sports for exercise, heavy gardening or similar ≥ 4 h a week	43		46		40	
Walking, cycling or light exercise ≥ 4 h a week	38		25		48	
Reading, watching television or doing other sedentary work/activity	2		2		3	
Education						
In the process of education	19		25		15	
Short education (12–13 years)*	17		17		16	
Medium higher education (15–17 years)	19		16		22	
Long higher education (+17 years)	45		42		47	

* Short education: no education after school, vocational training, shorter courses and short-term higher education.

### Reporting days

The distribution of 24 h DR by the day of the week is shown in Table 2. The dietary recalls were distributed fairly equally across all 7 d of the week.

**Table 2.** Distribution (number of days and percentage) of 24 h diet recall (24 h DR) by day of the week (*n* 358)

Day of the week	Number of days	Percentage
Monday	41	12
Tuesday	56	16
Wednesday	58	16
Thursday	46	13
Friday	48	13
Saturday	47	13
Sunday	62	17
Total	358*	100

* Two persons did not complete the last telephone interview. Three interviews were completed but lost due to a power breakdown.

### Energy intake *v*. energy expenditure

**Fig. 2.**
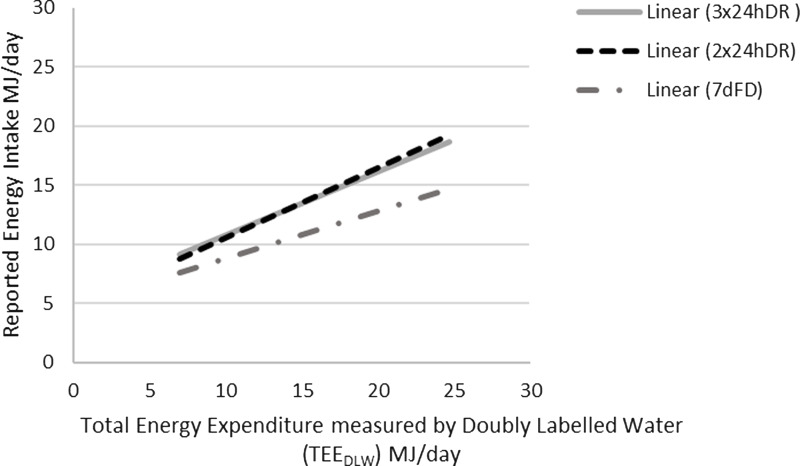
Linear relationship between total energy expenditure measured by the doubly labelled water (TEE_DLW_) and reported energy intake estimated by two (2 × 24 h DR) and three (3 × 24 h DR) 24 h diet recalls and a 7-d food diaries (7 d FD) (*n* 120).

The linear relationship between TEE and the reported EI by 24 h DR and 7 d FD is illustrated in Fig. 2.

The mean and mean difference of the reported EI by 7 d FD and 24 h DR compared with TEE_DLW_, and correlation coefficients for TEE_DLW_ and EI are shown in Table 3. Furthermore, participants’ weight change during the two dietary assessment periods, participants’ pedometer-determined PA (raw steps and steps including cycling) and correlation coefficients between Steps_CYCLING,_ time spent on MVPA during the last week and PAEE can be seen in Table 3.

**Table 3.** Mean daily energy measured by DLW and dietary assessment methods, weight change, and daily steps and self-reported time spent on moderate-to-vigorous physical activity (MVPA) the last week (mean values and standard deviations; 95 % confidence intervals)

	All (*n* 120)			Males (*n* 52)			Females (*n* 68)		
	Mean	95 % CI		Mean	95 % CI		Mean	95 % CI	
TEE_DLW_ (MJ/d)	11·5	11·0, 12·0		13·2	12·3, 14·0		10·2	9·8, 10·6	
7-d food diary (7 d FD); *n* 120									
Energy intake: EI_7 d FD_ (MJ/d)	9·5	9·0, 9·9		10·7	9·9, 11·40		8·5	8·07, 9·0	
Mean difference: EI_7 d FD_ – TEE_DLW_ † (MJ/d)	−2·0	−2·5, −1·5**		-2·5	−3·4, −1·6**		−1·7	−2·2, −1·1**	
Reported EI_7 d FR_: EI_7 d FD_/TEE_DLW_ %	84	80, 88		83	77, 90		85	80, 90	
Under-reported EI_7 d FR_: EI_7 d FD_- TEE_DLW/_TEE_DLW_ %	−16	−20, −12		−17	−23, −10		−15	−20, −10	
Pearson’s correlation: EI_7 d FD_:TEE_DLW_ ‡	0·44	0·29, 0·58**		0·34	0·07, 0·56*		0·16	−0·08, 0·39	
	Mean	SD		Mean	SD		Mean	SD	
7 d FD weight change (kg)	−0·1	0·9		−0·0	1·10		−0·1	0·8	
	Mean	95 % CI		Mean	95 % CI		Mean	95 % CI	
24 h diet recall (24 h DR); *n* 115									
Energy intake: EI_3 × 24 h DR_ (MJ/d)	11·6	11·0, 12·3		13·1	11·9, 14·2		10·6	9·0, 11·2	
	Mean	SD	95 % CI	Mean	SD	95 % CI	Mean	SD	95 % CI
EI_2 × 24 h DR_ (MJ/d)	11·5	3·7	10·8, 12·2	13·2	4·3	12·0, 14·4	10·2	2·6	9·6, 10·8
EI_2 × 24 h DR_ usual§ (MJ/d)	11·5	2·7	11·0, 12·0	13·1	2·9	12·3, 13·9	10·3	1·7	9·9, 10·7
	Mean	95 % CI		Mean	95 % CI		Mean	95 % CI	
EI 1st 24 h DR (MJ/d)	11·3	10·5, 12·1		13·4	12·0, 14·9		9·7	9·1, 10·4	
EI 2nd 24 h DR (MJ/d)	11·6	10·8, 12·5		12·9	11·4, 14·4		10·7	9·8, 11·6	
EI 3rd 24 h DR (MJ/d)	12·0	11·1, 12·8		12·8	11·4, 14·2		11·3	10·3, 12·3	
Mean difference EI_2 × 24 h DR_ – TEE_DLW_	0·0	−0·6, 0·6		-0·0	−1·3, 1·3		0·0	−0·6, 0·6	
Reported EI_2 × 24 DR_: EI_2 × 24 hDR_/TEE_DLW_ (%)	102	96, 108		103	93, 113		101	95, 108	
Under-reported EI_2 × 24 h DR_: EI_2 × 24 h DR_- TEE_DLW/_ TEE_DLW_ (%)	0·0	−0·0, 0·1		0·0	-0·1, 0·1		0·0	−0·1, 0·1	
Pearson’s correlation: EI_2 × 24 h DR :_TEE_DLW_	0·43**	0·28, 0·57		0·27*	−0·01, 0·50		0·33**	0·10, 0·53	
Pearson’s correlation: EI_3 × 24 h DR :_TEE_DLW_	0·44**	0·28, 0·58		0·34*	0·07, 0·56		0·24*	0·00, 0·46	
	Mean	SD		Mean	SD		Mean	SD	
24 h DR weight change (kg)	0·2	1·0		0·4	0·9		0·0	1·0	
7 d FD and 24 h DR weight change (kg)	0·4	1·1		0·4	1·3		0·4	1·0	
	Mean	95 % CI		Mean	95 % CI		Mean	95 % CI	
Physical activity									
Steps_RAW_ (Raw steps/d)	9236	8748, 9724		9312	8592, 10 032		9178	8500, 9856	
Steps_CYCLING_ (steps including cycling)	11 694	10 975, 12 413		11 821	10 739, 12 902		11 596	10 610, 12 582	
Pearson’s correlation: Steps_CYCLING_: PAEE‖	0·44	0·29, 0·58**		0·39	0·14, 0·60**		0·59	0·14, 0·60**	
Self-reported time (hours) spent on MVPA the last week	5·1	4·4, 5·9		6·0	4·9, 7·2		4·4	3·5, 5·4	
Spearman correlation: time spent on MVPA: PAEE‖	0·58	0·42, 0·68**		0·43	0·17, 0·64**		0·57	0·37, 0·72**	

* Significant at the 0·05 level (two-tailed).

† Paired sample *t*-test between reported energy intake and measured total energy expenditure.

‡ Pearson’s correlation coefficient.

§ EI estimate where intrapersonal (or day-to-day) variation from the group’s reported intake has been removed by the MSM method.

‖ PAEE = TEE_DLW_ – BMR calculated after Henry (2005) based on height and weight.

** Significant at the 0·01 level (two-tailed).

The mean reported EI by the 7 d FR and 2 × 24 h DR method was 9·5 MJ/d (95% CI (9·0, 9·9)) (males = 10·7 MJ/d; females = 8·5 MJ/d) and 11·5 MJ/d (95% CI (10·8, 12·2)) (males = 13·2 MJ/d; females = 10·2 MJ/d), respectively. 3 × 24 h DR provided a mean EI similar to the 2 × 24 h DR of 11·6 MJ/d (95% CI (11·0, 12·3)) (males = 13·1 MJ/d; females = 10·6 MJ/d). TEE_DLW_ was 11·5 MJ/d (males = 13·2 MJ/d; females = 10·2 MJ/d). Mean EI estimated by 7 d FR and 2 × 24 h DR were 84 % (males 83 %; females 85 %) and 102 % (males 103 %; females 101 %) of the TEE_DLW,_ respectively. There was a statistically significant difference between EI assessed by the 7 d FD and TEE_DLW_ (*P* < 0·01) but not between EI assessed by 2 × 24 h DR or 3 × 24 h DR and TEE_DLW._

All Pearson’s correlation coefficients were statistically significant for both dietary assessment methods and TEE_DLW_ (7 d FR *r* = 0·44 (95% CI (0·29, 0·58)); 2 × 24 h DR and 3 × 24 h DR *r* = 0·43 (95% CI (0·28, 0·57)) and 0·44 (95% CI (0·28, 0·58)) respectively), except for women using the 7 d FR method (*r* = 0·16). However, the strength of association was weak for both sexes regardless of the method. There were no statistically significant differences between correlation coefficients between 7 d FD and 2 × 24 h DR or 3 × 24 h DR, or between 2 × 24 h DR and 3 × 24 h DR; thus, the linear relationship between EI and TEE_DLW_ is comparable for the three methods.

**Fig. 3.**
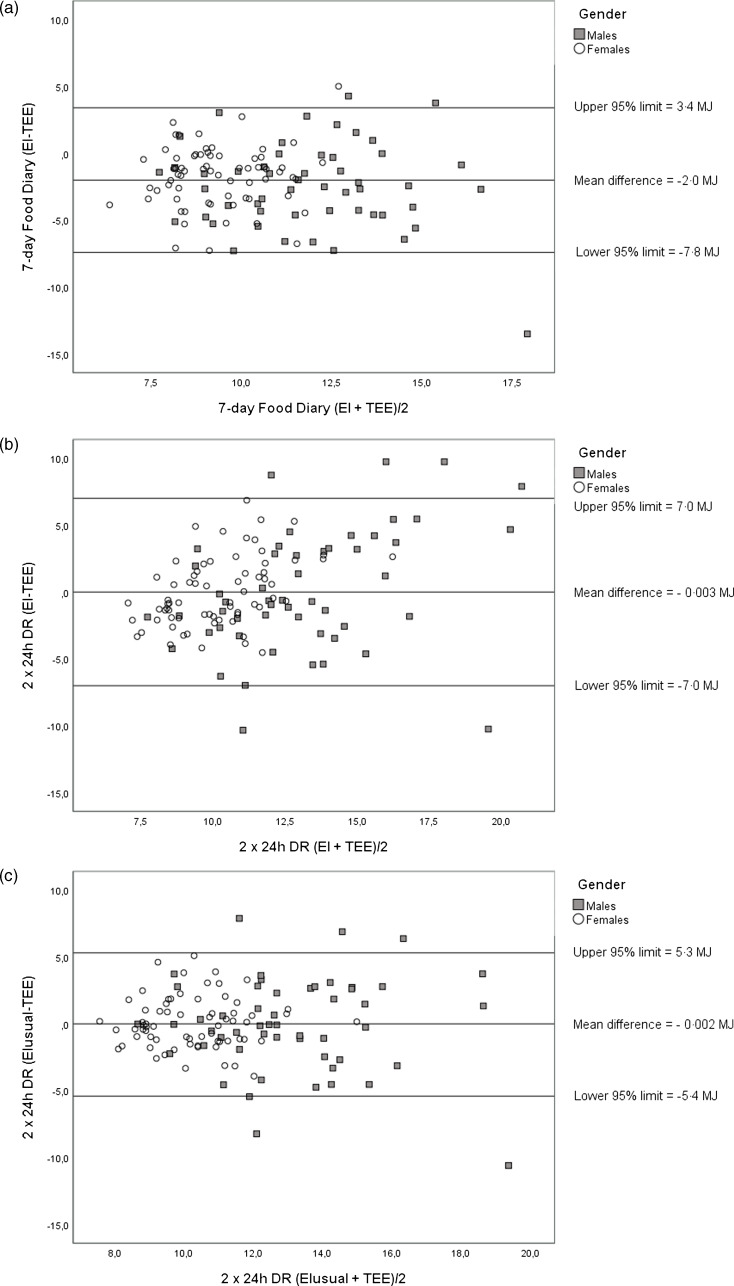
Difference between energy intakes (EI) calculated from the 7-d web-based food diary (7 d FD) (a), and the 2 × 24 h dietary recall (2 × 24 h DR) (b) and energy expenditure (TEE_DLW_) measured by the doubly labelled water method, plotted against the mean of the measurements EI and TEE. (b) The raw data from the 2 × 24 h DR. (c) The usual energy intake estimated by the multiple source method (MSM)^(28)^.

Bland–Altman limits of agreement were narrower for 7 d FR (Fig. 3(a)) than for the 2 × 24 h DR method (Fig. 3(b)). However, using usual intakes estimated from the 2 × 24 h DR MSM method, the limits of agreement were of the same magnitude as for the 7 d FD (Fig. 3(c)). For the 2 × 24 h DR, the agreement between EI and TEE_DLW_ varied with the magnitude of EI. However, this was less pronounced with usual intakes estimated by the 2 × 24 h DR MSM method. However, there was substantial error with both methods as limits of agreement were ± 50 % (± 5 MJ/d) of TEE_DLW_ for the 7 d FR and the adjusted 2 × 24 h DR.

There were no statistically significant weight changes in the participants during the study period with the two dietary assessment methods (Table 3).

### Physical activity

Result for PA is shown in Table 3. The mean measured Steps_CYCLING_ per day was 11 694 (95 % CI (10 975, 12 413)) (males = 11 821 steps/d; females = 11 596 steps/d). There was no statistically significant difference between males and females in steps per day. Pearson’s correlation coefficient between Steps_CYCLING_ and PAEE was overall *r* = 0·44 (males: *r* = 0·39; females: *r* = 0·59) (*P* < 0·01). Spearman’s correlation coefficients for self-reported time spent in MVPA were 0·58 *P* < 0·01 and statistically significant for both males and females (both *P* < 0·01).

### Reporting status

Table 4 shows that 61 %, 34 % and 5 % (7 d FR), 84 %, 4 % and 12 % (2 × 24 h DR) and 79 %, 6 % and 15 % (3 × 24 h DR) were classified as AER, UER and OER, respectively. More females than males were classified as AER with both the 7 d FR (65 % *v*. 56 %) and the 2 and 3 × 24 h DR (90 % *v*. 77 % and 82 % *v*. 75 %, respectively). There was no statistically significant difference between reporting status in relation to weight status for either of the methods.

**Table 4.** Percentage of under, acceptable and over-reporters* (Percentages)

	7 d FD			2 × 24 h DR			3 × 24 h DR		
	All	Males	Females	All	Males	Females	All	Males	Females
	%	%	%	%	%	%	%	%	%
Under-reporters	34	39	31	4	10	0	6	12	2
Acceptable-reporters	61	56	65	84	77	90	79	75	82
Over-reporters	5	6	4	12	14	10	15	14	16

* The accuracy of the reported EI was assessed using the confidence limits of agreement for EI/TEE_DLW_ as suggested by Black (10) 95 % CL = ± 2 × √(CV^2^_TEE_ + CV^2^_EI_/d).

Table 5 shows the cross-classification between TEE_DLW_ and 2 × 24 h DR, 3 × 24 h DR, 7 d FD, and PAEE and Steps_CYCLING_ and time spent in MVPA. Exact agreement was 51 % for the 7 d FD, 51 % for 2 × 24 h DR, 48 % for 3 × 24 h DR, 52 % for Steps_CYCLING_ and 57 % for time spent in MVPA. Percentages for participants classified in opposite tertiles were 7 % for the 7 d FD, 12 % for 2 × 24 h DR, 11 % for 3 × 24 h DR and 8 % for Steps_CYCLING,_ and 6 % time spent in MVPA.

**Table 5.** Percentages of participants classified in the same, adjacent and opposite tertiles of total energy expenditure measured by doubly labelled water (TEE_DLW_), energy intake (EI), steps including cycling (Steps_CYCLING_) and time spent on moderate-to-vigorous physical activity (MVPA) the last week

	Exact agreement (%)	Adjacent tertile (%)	Opposite tertile (%)	*κ* (*κ*_w_)
EI_7 d FD_ *v*. TEE_DLW_	51	42	7	0·26**
EI_2 × 24 h DR_ *v*. TEE_DLW_	51	37	12	0·26**
EI_3 × 24 h DR_ *v*. TEE_DLW_	48	41	11	0·22**
Steps_CYCLING_ *v*. PAEE†	53	49	8	0·30**
MVPA *v*. PAEE†	57	38	6	0·35**

† PAEE = TEE_DLW_ – BMR calculated after Henry based on height and weight.

** Significant at the 0·01 level.

### Acceptability and preference of the two dietary assessment methods

As illustrated in Table 6, 77 % preferred the 7 d FD and 23 % preferred the 3 × 24 h DR. The 7 d FD was evaluated as more flexible, enabling diet registration whenever participants had the time. The 3 × 24 h DR was preferred because it was faster and easier because the interviewer completed the diet registration. Eighty-three percentage believed that the 7 d FD was the best method for capturing their dietary intake as it covered the whole week. Seventeen percentage thought it was the 2 × 24 h DR method as it gave a more detailed and precise description of the foods and beverages reported. In the focus groups, other issues were revealed, especially that the 7 d FD affected the participants’ diet. Participants failed to eat food that was cumbersome to register (street food was mentioned as an example) or they forgot to register taking second portions. Participants also stated that they became much more aware of what they were eating and drinking and may have changed their intake in a healthier direction with the 7 d DR. One participant mentioned that the 24 h DR also influenced her reporting because she had to tell the interviewer what she had eaten.

**Table 6.** Preferred method

	7-d web-based FD	3 × 24 h DR and FFQ
Preferred method (%)	77	23
Reasons	More flexible and convenient (can do it when you have time)	Easier and faster (interviewer does the registration)
Best method to capture dietary habits (%)	83	17
Reasons	7 d (a whole week) is more accurate than 2 or 3 d	Gives a more precise description of the food eaten including preparation. More foods and beverages reported

For the 24 h DR, it was especially the memory of food intake and portion sizes that was evaluated as difficult. Therefore, participants were happy to have the interview appointments beforehand, so they could quickly recall what they had eaten the day before – before the interviewer called. Participants felt that the questioning and probing throughout the interview, especially about details, made them report more foods and details such as butter on bread and sugar in coffee during the 24 h DR than in the7 d FD reporting. The FMB was seen as awkward because it had an abnormal size. Some thought it was difficult to use for portion size estimation. The mounds on the plate and the grid model were not easy to use; it was easier with realistic pictures, as in the 7 d FD instead of arbitrary forms. However, it was helpful that the forms were real sizes.

Participants evaluated both methods as both cumbersome and easy. The 7 d FD was evaluated as cumbersome because it had to be completed every day for 7 consecutive days with searching, clicking and writing, and the 3 × 24 h DR because of the fixed times for the interviews and the long interview with repetitive questions. The interviewers also experienced that some participants were annoyed by the repetitive questions, but they also experienced that many participants came up with more foods eaten when asked about often-forgotten foods and food and drinks between meals. In general, the interviewers experienced that the participants had difficulties using the FMB because of the many options and the scrolling needed to find the most suitable model.

## Discussion

We examined the agreement between EI estimated by the 7 d FD and 24 h DR – dietary assessment methods widely used in national dietary surveys – and TEE_DLW_ measured by the reference DLW method. In a weight-stable sample of volunteers 18–60 years, EI estimated with both 2 × 24 h DR and 3 × 24 h DR showed no absolute differences with TEE_DLW_. The EI calculated using the 2 × 24 h DR were not underestimated while the 7 d FD underestimated EI by on average -16 %. In a Swedish study validating a 4 d FD against TEE_DLW_ in forty males and females aged 50–64 years, they found that EI was underestimated by -20 %^(30)^, which is a little higher than in our 7 d FD. In the validation study of 3 × 24 h DR using the AMPM against TEE_DLW_ by Moshfegh *et al.* among 524 males and females aged 30–69 years, they found that EI was underestimated by -11 % overall^(13)^. In a Norwegian study, a three step interviewer-administered, computer-based 4 × 24 h DR by telephone underestimated EI by -17 % when compared with TEE_DLW_ in 18–70-year-old participants^(31)^. This is higher than the findings in the present study and in the study by Moshfegh *et al.* The difference may be explained by the interviewing technique and the degree to which participants were questioned about foods they may have forgotten or overlooked. The Norwegian study did not include a first in-person interview and included a three-step method, whereas the AMPM includes a five-step approach including more questions and opportunities to report forgotten foods.

In a Brazilian study validating both a 2 d FD and the 3 × 24 h DR (also AMPM) among eighty-three volunteers aged 20–60 years, they found that EI was underestimated by −28 and −33 % for the two dietary assessment methods, respectively^(32)^. This is considerably higher than in the present study. This might be because of a lower educational level in the study of Lopes *et al*., or another food culture with more unplanned eating, which is harder to remember. In a recent review by Burrows *et al*. including fifty-nine studies validating estimated EI with different dietary assessment methods by TEE_DLW_, they also found that under-reporting was highly variable within studies and between dietary assessment methods, with 24 h DR having less variation and degree of under-reporting compared with other assessment methods^(33)^.

In the present study, 61 % of the participants were classified as AER, 34 % as UER and 5 % as OER with the 7 d DR. In the study by Nybacka *et al*., 55 % were classified as AER, 40 % as UER and 5 % as OER, which is similar to the 7 d FD in the present study^(30)^. In comparison, 84 % and 79 % were classified as AER, 4 % and 6 % as UER and 12 % and 15 % as OER with the 2 × 24 h DR and 3 × 24 h DR in the present study, respectively. In a review by Livingstone, they also found high proportions of UER by both validated dietary assessment methods ranging from 17 to 59 % with the FD and smaller proportions ranging from 12 to 46 % for the 24 h DR^(34)^.

In the study by Mosfegh *et al*.^(13)^, a similar proportion of AER were found, but more UER and fewer OER. The difference in distribution of UER and OER between the present study and the study by Moesfegh *et al*. can also be due to the estimation of TEE_DLW._ In the present study, newer equations published by Speakman were used to calculate rates of CO_2_ production^(21)^, whereas in the study by Moesfegh *et al*. they used other equations for estimating TEE_DLW_^(35,36)^. We also calculated TEE_DLW_ using the Scholler equations^(37,38)^ and found it gave higher TEE_DLW_ estimates (around 0·5 MJ/d), and thereby fewer OER and more UER.

Although there was good agreement between EI and TEE_DLW_ with especially the 2 × 24 h DR and 3 × 24 h DR at group level, the data showed substantial variability in the accuracy of both methods at the individual level. EI measured by the 7 d FD showed narrower limits of agreement estimated by the Bland–Altman method than the 2 × 24 h DR, and similar to those of Nybacka *et al*.^(30)^ However, when using the usual intake estimates from the 2 × 24 h DR MSM method, the limits of agreement were of the same magnitude as for the 7 d FD. In the Brazilian study by Lopes *et al.*, wide limits of agreement for both methods were also found, probably because of the few reporting days (2 with the FD)^(32)^. From the Bland–Altman plot, we found that the agreement between EI and TEE_DLW_ tended to vary with the magnitude (higher EI and/or TEE_DLW_) for the 2 × 24 h DR, but this was not pronounced using the estimated usual intakes from the 2 × 24 h DR by the MSM method. This is because the MSM method significantly improves the estimation of the tails of the intake distribution when compared with the traditional method^(39)^. However, in both the present study, and in the study by Nybacka *et al*.^(30)^ and Lopes *et al*.^(32)^ referred to above, the limits of agreement are large corresponding to ± 50–60 % of TEE_DLW_, and hence the measurement error is substantial.

The ability to rank individuals according to EI *v*. TEE_DLW_ was evaluated by Pearson’s correlations and cross-classifications. In the present study, the correlation coefficient was *r* = 0·44 for the 7 d FD and *r* = 0·43 and 0·44 for the 2 × 24 h DR and 3 × 24 h DR, respectively. This is in line with the findings in the study by Nybacka *et al*. where correlation coefficient of *r* = 0·40 for the 4 d FD was found^(30)^. In a Norwegian study validating 4 × 24 h DR, the deattenuated Pearson’s correlation coefficient was *r* = 0·34^(31)^. This is in line with the review of Burrows *et al.* where both lower and higher correlation coefficients were reported for both validated dietary assessment methods. Most studies ranged from *r* = 0·19–0·79 for FD and *r* = 0·22–0·64 for 24 h DR^(33)^. However, when the EI of males and females is correlated together, higher correlation coefficients will be generated, because males’ higher EI contributes to a positive correlation slope. Therefore, the sex-specific correlation coefficients give better figures for the ability to rank individuals. With the 7 d FD, correlations for females were non-significant, and with the 2 × 24 h DR and 3 × 24 h DR correlation coefficients were significant for both males and females.

The weighted *κ* analysis showed that 48–51 % were classified in the same tertile of TEE_DLW_ and 7 d FD, 2 × 24 h DR and 3 × 24 h DR. Only few studies display cross-classification results and k-statistics, probably due to the low number of participants in validation studies using the DLW technique. But our results are comparable with the findings in the study of Nybacka *et al*. for the 4 d DR, showing that 48 % were classified in the same tertile as TEE_DLW_.

Adding the third recall did not seem to improve the ranking ability of EI estimated by 2 × 24 h DR in the correlations and cross-classification. Adding a third recall tended to increase over-reporting (from 12 % to 15 %). Based on remarks from interviewers, a possible explanation could be due to an exhaustion effect at the 3rd recall, and that participants did not take so much care in reporting corrections to the recall and leftovers to shorten the interview. A third recall, however, most likely improves the accuracy for intake of nutrients and food groups due to the large intra-variability in participants intake with only two recalls.

### Acceptability

The validity and feasibility of self-reported dietary intake are influenced by the ways participants respond to the dietary assessment methods. In the present study, participants preferred the 7 d FD because they perceived it as more flexible allowing them to report whenever they had time. The covering of a whole week was seen as giving a more accurate picture of the dietary habits, despite participant was aware of simplifying their food intake, eating less than usual and healthier. Participants felt that the 24 h DR was unfair because it was based on fewer reporting days, days that may not represent ‘normal’ days, due to, for example, attending a party, conference, etc. – even if participants stated that with the 24 h DR they were able to report their intake more precisely and that the assessment method most likely prompted them to report more of the food and beverages they forgot in the 7 d FD. In an American study evaluating participants’ experiences with FD in focus groups, it was also found that participants experienced they simplified their diet. Participants reported consuming simpler foods, foods in predefined portion sizes, ate less frequently, ate fewer snacks and were not eating at restaurants because of the hassle of recording every item^(40)^. In another study by Silveria *et al.*, they evaluated the acceptability of completing six self-administered 24 h DR using the web application ASA24, by means of a questionnaire. Here, participants experienced that it was hard to remember everything eaten the previous day^(41)^. In an English study evaluating four dietary assessment methods by low-income households, the participants preferred a 4-d food checklist with pre-coded answers and space for reporting ‘other foods’ using standard portions or self-reported portions compared with 4 × 24 h DR, a semi-weighed method and a weighed inventory. However, interviewers preferred the 24 h DR method because more foods and details were reported^(42)^. In the present study, interviewers also experienced participants reported more of their food intake with the 24 h DR method.

Thus, previous research supports the findings in the present study that the 7 d FD leads to more simplified dietary habits and consuming less and probably more healthful foods. This was also reflected in the proportion of UER. For the 24 h DR, it is the memory of previous days’ intake and especially the portion size estimation that is the main reason for misreporting. Even if participants knew the time for the telephone interviews and did some recapturing of the previous 24 h food intake, it was evaluated as difficult. The awareness of the telephone interview and the recapturing of intake could also affect the reported intake in a healthier direction. However, it would be unrealistic with unscheduled interviews, because of the need of participants time for the lengthy 24 h DR and the requirement to cover all weekdays equally.

### Physical activity

In the present study, we also evaluated Steps_CYCLING_ and self-reported MVPA as measures for the participants’ PA against PAEE. Pearson’s correlation coefficients for Steps_CYCLING_ were significantly correlated at the same level overall as the 7 d FD, 3 × 24 h DR and TEE_DLW_. However, correlation coefficients for daily steps were stronger for both males and females (*P* < 0·01). The relationship between pedometer-determined PA and TEE is complicated by the fact that a step is a movement, whereas TEE also reflects effect of sex, age and BMI in addition to movement and efficiency of movement. Tudor-Locke *et al.* reported that pedometers correlate moderately with different measures of EE (heart rate estimated EE, indirect calorimetry and TEE_DLW_) (range = 0·46–0·88)^(43)^. For TEE_DLW_, both significant and non-significant correlations were shown. In the present study, cross-classification of Steps_CYCLING_ against PAEE showed that 52 % were classified in the same tertile and significant k-statistics. This is at the same level as for the 7 d FD and the 24 h DR.

In the present study, the Spearman correlation for self-reported time spent in MVPA and PAEE showed a moderate overall significant correlation of *r* = 0·58 and significant correlation coefficients for both males and females. The cross-classification showed that 57 % were classified in the same tertile of PAEE and MVPA.

Overall, the above results indicate that both pedometer-determined steps and self-reported time spent in MVPA can be used as predictors of PAEE in adult Danes.

### Strengths and limitations

The strength of the present study is the large study sample of validating EI against TEE_DLW._ Similar studies often include fewer participants^(33)^. Another strength is that there were no dropouts in the present study. Only two participants were not able to complete the last 24 h DR. The low dropout could be due to the study population comprising motivated, higher educated and health interested volunteers.

There are several limitations to this study. First, we used a TEE_DLW_ CV derived from another population^(23)^ to estimate the confidence limits of agreement for EI/TEE_DLW_ to define reporting status, because the DLW was applied only once in all participants. This procedure, however, has been used in several studies, probably due to the high cost and participant burden of repeating the DLW technique^(30,32)^.

Another possible limitation in this study is the short-time interval (approximately 6 d) between the two dietary assessments. This could influence the dietary assessment in several ways; by a decline in the quality of dietary reporting due to the study load resulting in poorer dietary assessment with the last method or altered eating habits because of the continued awareness of own dietary habits or to avoid the hassle of reporting. It could also result in better reporting with the last method because of the training practice, for example, remembering all the details asked about in the 24 h DR, and therefore being more attentive to the last method, if this was the 7 d FD. It was not practical to prolong the study period because it was considered important that the DLW covered as much of the dietary and PA assessment period as possible, and this was considered the best way. However, to account for the carry-over effect between dietary assessment methods and make this equal for both methods, we applied a cross-over design. Furthermore, there was no significant difference in EI depending on the administration order of the assessment methods.

Finally, the high proportion of volunteer participants with longer education (45 % *v*. 14 % in the population) limits the generalisability of the present results. Analysis by education indicated that participants with short education produced similar results. However, the study did not adequately cover people with no or vocational education. Furthermore, educational groups were too small to make strong statistical comparisons.

### Conclusion

The validity of two of the most commonly used dietary assessment methods in national dietary surveys was examined by comparing EI to TEE_DLW_ in a volunteer Danish adult population. The accuracy of reported mean EI at group level was higher with the 2 × 24 h DR than for the 7 d FD among adult Danes. EI was underestimated by the 7 d FR by −16 % but not underestimated with the 2 × 24 h DR. There was substantial variability in the accuracy of the dietary assessment methods at the individual level for both methods. Under-reporting is a major concern with the 7 d FD where participants themselves report that the method contributes to altered dietary habits. With the 24 h DR, it is most likely memory that causes misreporting. Adding a third recall to the 2 × 24 h DR method did not seem to improve the ranking of individuals’ EI. Viewed from a point of accuracy of assessed EI on group level, the 2 × 24 h DR method should be recommended. But other factors such as estimation of food groups and nutrients as well as acceptance should also be taken into consideration. Pedometer-determined PA including cycling (Steps_CYCLING_) and self-reported time spent in MVPA also showed ability to rank individuals according to PAEE and can be considered as predictors of PAEE in adult Danes.
