# Validation of the 2 × 24 h recall method and a 7-d web-based food diary against doubly labelled water in Danish adults – CORRIGENDUM

**DOI:** 10.1017/S0007114524001156

**Published:** 2024-08-14

**Authors:** Anja Biltoft-Jensen, Karin Hess Ygil, Lenette Knudsen, Jeppe Matthiessen, Sisse Fagt, Ellen Trolle, Trine Holmgaard Nielsen, Diane McIntosh Hansen, Cecilie Löe Licht, Maurice Martens, Catherine Hambly, John R. Speakman, Tue Christensen

The author regrets the incorrect allocation of author affiliations in the published article. The affiliations were given as:

Anja Biltoft-Jensen^1^*, Karin Hess Ygil^1^, Lenette Knudsen^2^, Jeppe Matthiessen^1^, Sisse Fagt^1^, Ellen Trolle^1^, Trine Holmgaard Nielsen^3^, Diane McIntosh Hansen^4^, Cecilie Löe Licht^5^, Maurice Martens^6^, Catherine Hambly^7^, John R. Speakman^7,8^ and Tue Christensen^1^

^1^National Food Institute, Technical University of Denmark, Kongens, Lyngby 2800, Denmark

^2^Steno Diabetes Center Copenhagen, Region Hovedstaden, Gentofte, Denmark

^3^Danish Cancer Society, Counselling Centre Herlev, Herlev, Denmark

^4^Kalvebod Fælled School, København S, Denmark

^5^Center for Cancer Immune Therapy (CCIT), Department of Oncology, Herlev Hospital, Herlev, Denmark

^6^Centerdata, Tilburg University, Tilburg, Netherlands

^7^Institute of Biological and Environmental Sciences, University of Aberdeen, Aberdeen, Scotland, UK

^8^Shenzhen Key Laboratory of Metabolic Health, Center for Energy Metabolism and Reproduction, Shenzhen Institutes of Advanced Technology, Chinese Academy of Sciences, Shenzhen, People’s Republic of China

The correct affiliations are:

Anja Biltoft-Jensen^1^*, Karin Hess Ygil^1^, Lenette Knudsen^1^, Jeppe Matthiessen^1^, Sisse Fagt^1^, Ellen Trolle^1^, Trine Holmgaard Nielsen^1^, Diane McIntosh Hansen^1^, Cecilie Löe Licht^1^, Maurice Martens^2^, Catherine Hambly^3^, John R. Speakman^3,4^ and Tue Christensen^1^

^1^National Food Institute, Technical University of Denmark, Kongens, Lyngby 2800, Denmark

^2^Centerdata, Tilburg University, Tilburg, Netherlands

^3^Institute of Biological and Environmental Sciences, University of Aberdeen, Aberdeen, Scotland, UK

^4^Shenzhen Key Laboratory of Metabolic Health, Center for Energy Metabolism and Reproduction, Shenzhen Institutes of Advanced Technology, Chinese Academy of Sciences, Shenzhen, People’s Republic of China

The article has been corrected. The author apologises for the error.
